# EMP3 Expression in HER2‐Enriched Breast Cancer is Linked to PI3K/AKT Signaling and Indicates Poor Prognosis

**DOI:** 10.1155/genr/8242640

**Published:** 2026-04-22

**Authors:** Mingda Zhu, Chenyang Tian, Ninine Marthe Nkenfag, Niannian Wang, Weiping Yu

**Affiliations:** ^1^ Shanghai Pudong Hospital, Fudan University Pudong Medical Center, 2800 Gongwei Road, Pudong, Shanghai, 201399, China, fudan.edu.cn; ^2^ Department of Thoracic Surgery, Section of Esophageal Surgery, Shanghai Chest Hospital, Shanghai Jiao Tong University School of Medicine, Shanghai, 200030, China, shsmu.edu.cn; ^3^ Department of Breast, Affiliated Cancer Hospital of Zhengzhou University & Henan Cancer Hospital, Zhengzhou, Henan, China, zzu.edu.cn

**Keywords:** EMP3, HER2-enriched breast cancer, PI3K–AKT, poor outcomes

## Abstract

**Purpose:**

EMP3 is closely associated with HER2 expression and trastuzumab resistance. However, the prognostic value of EMP3 in the HER2 subtype remains poorly understood. It is necessary to clarify the relationship between EMP3 and prognosis in HER2‐enriched breast cancer.

**Methods:**

The expression of EMP3 was measured using immunohistochemistry staining. Then, Chi‐square tests were carried out to investigate the relationship between EMP3 and relevant clinical data. After that, univariable and multivariable Cox regression were used to filter out factors with independent prognostic value, while a nomogram was constructed to forecast the disease‐free survival (DFS) rates of patients. Subsequently, we employed R to identify genes exhibiting a correlation coefficient exceeding 0.6 with EMP3 expression and then mapped such genes onto the PI3K–AKT pathway through the KEGG database. Patients were then stratified into two subgroups based on the median EMP3 expression, and differential expression status of the PI3K–AKT pathway between the groups was analyzed. The proliferation capacity under trastuzumab treatment was detected through plate colony formation and EdU incorporation assays in cells with different EMP3 expression levels. Nude mouse xenograft tumor formation experiments were employed to further verify the connection between EMP3 expression and trastuzumab resistance.

**Results:**

A total of 90 HER2‐enriched breast cancer patients were enrolled in this research. EMP3 expression did not exhibit any significant association with age, menopausal status, T‐stage, N‐stage, androgen receptor, Ki67, EGFR, or CK5/6. Results from univariate and multivariate Cox regression analysis confirmed that EMP3 expression, T‐stage, and N‐stage served as independent prognostic factors for DFS. The prognostic model constructed based on EMP3 expression, T‐stage, and N‐stage has good predictive power. The model’s *C*‐index was measured to be 0.745. The calibration curves of the model largely coincided with the diagonal line, suggesting that the actual DFS of patients was almost the same as the DFS predicted by the model. The genes positively associated with EMP3 expression status bear a close association with the PI3K–AKT pathway among HER2‐enriched breast cancers. Enhanced activation of the PI3K–AKT pathway was detected in the EMP3 high‐expression group. Results derived from both in vitro and in vivo experiments demonstrate that EMP3 regulates the PI3K/AKT pathway and promotes resistance to trastuzumab.

**Conclusions:**

High EMP3 expression in HER2‐enriched breast cancer predicts poor patient outcomes. Overexpression of EMP3 exerts an upregulatory effect on the PI3K–AKT pathway and boosts the development of trastuzumab resistance.

## 1. Background

Human epidermal growth factor receptor 2 (HER2)‐enriched breast cancer makes up 15%–20% of all breast cancers, with its key feature being the overexpression of the HER2 [1]. In comparison to other subtypes of it, like Luminal B and Luminal A, this particular subtype presents a more aggressive clinical course, along with elevated incidences of local recurrence and distant metastasis to sites such as the brain and bone; these two manifestations predict death in the short term [2]. Concerning systemic therapy, targeted treatment is the powerful therapeutic tool against this breast cancer subtype, while targeted therapy still has the problem of initial and acquired drug resistance. In theory, the most efficient approach to boost patient prognosis is to develop new targeted drugs or combined targeted therapy.

Trastuzumab (or Herceptin), a recombinant humanized monoclonal antibody, is a representative drug targeting HER2‐positive breast cancer. It can selectively play an antitumor role in HER2‐amplified breast cancer tissue but has no effect on HER2‐normally expressed tumors [3–5]. Unfortunately, in clinical practice, merely one‐third of patients with HER2‐positive have major reactions to trastuzumab, indicating that these individuals are initially or inherently resistant to trastuzumab [6, 7]. In addition, about 70% of women with HER2+ metastatic breast cancer (MBC) who had favorable initial responses to trastuzumab developed disease progression within 1 year, indicating that they had secondary or acquired drug resistance to trastuzumab [8]. At present, the drug resistance mechanisms of trastuzumab include (1) impaired binding to HER2 [9–12]; (2) HER2 mutations [13]; (3) cross‐talk between HER2 and estrogen receptors [14–17]; (4) enhanced signaling through HER2 and other receptors [18–20]; (5) downstream pathway activation [21, 22]; (6) inability to induce antibody‐dependent cell‐mediated cytotoxicity [23, 24]; (7) HER2 heterogeneity and differences in the degree of HER2 expression [25, 26], and so on. However, in HER2‐enriched breast cancer, the influence of crosstalk between HER2 and epithelial membrane protein (EMP) on drug resistance has long been overlooked, a situation that logically stems from the intricate nature of the crosstalk mechanism. Recent studies have identified a robust association between EMP3 and HER2—both members of the EMP family—highlighting their potential cooperative role in tumor progression.

Epithelial membrane protein 3 (EMP3) falls into the category of the peripheral myelin protein 22‐kDa (PMP22) gene family. It has a molecular weight of 18,429 kDa and is classified as a multipass membrane protein, equipped with four transmembrane domains as well as two predicted N‐linked glycosylation sites. Studies indicate that EMP3 mRNA expression is markedly elevated in primary tumor (breast cancer) compared to normal tissue, and its overexpression is strongly associated with high HER‐2 expression levels [27, 28]. Wang et al. observed that bladder cancer cell lines with stable HER2 overexpression exhibited significantly increased EMP3 expression relative to their parental lines. In TSGH8301/EMP3 cells, the expression of EMP3 was also inhibited when HER2 was inhibited by shRNA. Conversely, knockdown EMP3 also inhibited HER2. When TSGH8301/EMP3 cells were treated with trastuzumab, the expression of EMP3 was inhibited and HER2 expression was inhibited in a dose‐dependent manner. Importantly, EMP3 overexpression led to the development of trastuzumab resistance. These results strongly suggest a functional interplay between EMP3 and HER2 in vitro, indicating that EMP3 may play a significant role in the process of trastuzumab resistance [29]. Breast cancer‐related studies also draw a similar conclusion, that is, EMP3 is a significantly upregulated gene in the microarray analysis related to HER2 overexpression [30]. Therefore, EMP3 is an innovative co‐targeting candidate in the design of HER2‐positive cancer therapy.

However, the function of EMP3 in the HER2‐enriched subtype is unclear. Zhou et al. observed that EMP3 is capable of negatively regulating DNA replication processes, the repair of DNA damage, and the stem cell‐like traits of breast cancer cells. Therefore, EMP3 might function as a tumor suppressor gene during the occurrence and progression of breast cancer. However, there is no proliferation and cloning test related to the SK‐BR‐3 cell line in this article [31]. Interestingly, Hong et al. found that knockdown of EMP3 in the SK‐BR‐3 cell line can inhibit tumor migration and invasion [32]. Cha and Koo found in a clinical retrospective study that HER2‐positive was related to the expression of EMP3, but because the number of HER2‐enriched cases was too small, the two groups (EMP3‐positive and EMP3‐negative) showed no statistically significant differences in terms of overall survival (OS) and disease‐free survival (DFS). [33]. To comprehensively evaluate the prognostic significance of EMP3—particularly in patients with HER2‐enriched breast cancer—we investigated associations between EMP3 expression levels and key clinicopathological features as well as long‐term survival outcomes. Finally, we conducted enrichment analysis of genes positively expressed with EMP3 to explore the potential mechanism of trastuzumab resistance induced by EMP3.

## 2. Methods and Materials

### 2.1. Patient in This Study

From January 1, 2017, to December 31, 2017, tumors (primary) were obtained from 90 HER2‐enriched breast cancer patients treated at Zhengzhou University Henan Cancer Hospital. Every patient involved in the study provided written informed consent regarding the utilization of their personal data and tissue specimens.

### 2.2. Clinicopathological Data

The pathological data of all 90 patients included in this study were retrospectively collected from the patient database. All tissue samples underwent fixation with 10% neutral buffered formalin, were cut into 5‐μm thick slides, and had immunohistochemistry (IHC) performed according to standard protocol to clarify the expression statuses of ER, PR, HER2, AR, Ki67, CK5/6, and EGFR. The results were confirmed by two blinded pathologists. Positivity for each one of them was defined by the criteria below: (i) Positivity for progesterone receptor and estrogen receptor was identified in at least 1% of tumor cells via IHC; positivity of either one or both was considered hormone receptor (HR) positive, and the negativity of either one of them was considered HR‐negative. (ii) HER2 positivity was determined as a score of 3+ on the IHC assay or 2+ (equivocal) with HER2 gene amplification through fluorescence in situ hybridization (FISH). (iii) Androgen receptor (AR) positivity was identified when 10% or more of tumor cells showed positive nuclear stain on IHC. (iv) Ten high‐power visual fields were chosen for every tissue section, and the average proportion of Ki67‐positive cells relative to the total cell count was taken as a quantitative index. The threshold for Ki67 expression was 30% (< 30%, low expression; ≥ 30%, high expression). (v) CK5/6 and EGFR were defined as either positive or negative based on the tumor cells showing nuclear stain on IHC. Additional clinical variables such as age at diagnosis, menopausal status, tumor dimensions, and lymph node involvement status were subjected to statistical analysis.

### 2.3. Follow‐Up

This work was finalized on December 31, 2021. The median follow‐up duration reached 54 months with an interquartile range spanning from 46 to 54 months. We defined DFS as the time interval from the initial diagnosis to the first occurrence of tumor recurrence, detection of contralateral breast cancer, or identification of first distant metastasis. For patients without any related clinical events, data censoring was performed based on their last follow‐up time point.

### 2.4. IHC Staining and Scoring

Using patients’ pathological identity numbers, FFPE tissue blocks from the 90 patients in this study were retrieved from the pathology laboratory. Care was taken to retrieve the same FFPE tissue blocks from which previous pathological data were generated. We purchased the EMP3 antibody (catalog no. 30021; Signaling Antibody LLC, College Park, MD, USA).

Tissue sections were sliced from the FFPE tissue blocks (thickness 5 μm). After deparaffinization in xylene and sequential rehydration through a graded ethanol series (100%, 95%, 75%, and 50% v/v), immunohistochemical staining was performed using the streptavidin–peroxidase (SP) method according to the manufacturer’s instructions. For antigen retrieval, the tissue specimens were immersed in citrate buffer (pH 6.0), heated to a constant temperature of 100°C, and rinsed three times with PBS, with each rinse lasting. To inhibit the activity of endogenous peroxidase, the tissue sections were incubated with 3% hydrogen peroxide solution at room temperature under light‐free conditions for 25 min. After which, the tissues were rinsed 3 times for 5 min each with PBST buffer. A tissue section was subsequently treated with 10% bovine calf serum and incubated (room temperature for 15 min). Once the serum had dried up, the EMP3 primary antibody (used at a dilution ratio of 1:500) was added, and the sections were placed in a 4°C environment for overnight incubation. PBST was used to rinse the tissue Section 3 times for 5 min each. An antirabbit secondary antibody was subsequently applied and incubated, after which DAB chromogenic solution was applied for 5 min, followed by redyeing with hematoxylin for 90 min at room temperature, conventional dehydration, and sealing.

Researchers performed microscopic evaluations of EMP3’s staining intensity, positive expression range, and subcellular distribution via a bright‐field upright optical microscope (Olympus Corporation) under 200x and 400x magnification. For the quantification of EMP3 expression levels, the IHC sections were scored by calculating the staining intensity (0: no staining; 1: weak; 2: moderate; 3: strong) and staining proportion score (0%: negative; 1: < 30% positive; 2: ≥ 30% positive). Combined scores between 0 and 2 were regarded as negative for EMP3, whereas scores of 3 and above were considered positive.

### 2.5. Cell Lines and Cell Culture

JIMT‐1 (RRID: CVCL_1598) was cultured in Ham’s F‐12/DMEM (1:1) medium enriched with 10% fetal bovine serum. The SKBR‐3 (RRID:CVCL_0033) was acquired from the American Type Culture Collection and cultured following the supplier’s guidelines.

### 2.6. EMP3 Knockdown and Overexpression Experiments

The siRNA for EMP3 knockdown and the overexpression plasmid were purchased from Shanghai Jinghe Biotechnology Co., Ltd. The siRNA sequence is 5′‐CCC​TTC​ACA​TCC​TCA​TTC​TTA​TA‐3′. Human EMP3 gene sequences (NM_001425.3) were amplified and subsequently inserted into the GV219 plasmid, supplied by Shanghai Genechem Co., Ltd. Cells were transfected with plasmids by Lipofectamine 3000 (Invitrogen). Then, the changes in protein expression were detected by WB.

### 2.7. Colony‐Forming Experiment

Approximately 1000 cells were seeded per well and cultured for 14 days, or until the number of cells in the majority of single colonies exceeded 50, in DMEM supplemented with 10% FBS; the medium was refreshed every three days during this period. Following incubation, the cells were fixed with methanol and stained using crystal violet reagent (Solarbio, China), followed by manual enumeration and imaging of observable colonies. The total number of colonies was quantified with the aid of ImageJ (Version 1.52a). This procedure was performed independently for a minimum of three times, with each repetition including technical triplicates.

### 2.8. EdU Proliferation Detection Experiment

The Click‐iT EdU‐555 Cell Proliferation Assay Kit (product code: G1602, Servicebio, China) was employed to detect cellular proliferation activity. Cells were uniformly seeded at a defined density in 6‐well plates. After cell attachment, trastuzumab was added to the culture system, and the cells were maintained in incubation for 48 h. Subsequently, the proliferation assay was carried out following the operational guidelines provided by the kit manufacturer.

### 2.9. Xenograft Tumors

Female nude mice, aged 6 weeks, were obtained from Shanghai Jingshan Experimental Animal Co., Ltd. and subcutaneously injected with 2 × 10^6^ cells. Thirty mice were randomly assigned to two experimental groups. We assigned SK‐BR‐3 cells for inoculation in one group and designated JIMT‐1 cells as the inoculation material for the other group. Each group comprised five subgroups: blank control (*n* = 3), knockdown vector control (*n* = 3), gene knockdown (*n* = 3), overexpression vector control (*n* = 3), and gene overexpression (*n* = 3). Trastuzumab was delivered via intraperitoneal injection at a dosage of 5 μg/g, with administration conducted once every 4 days. The control group mice received intraperitoneal injections of 100 μL of normal saline at each administration. The volume of each tumor was calculated by multiplying its measured length, width, and height, measured every 4 days using a caliper. Animals were euthanized by CO_2_ inhalation in accordance with approved animal welfare guidelines.

The Ethics Committee (Zhengzhou University) granted approval for all the experimental protocols designed for this study. All operations were strictly executed following the relevant guidelines and regulatory provisions. We further confirm that the reporting of all research methods in this study complies fully with the requirements of the ARRIVE guidelines.

### 2.10. Bioinformatics Analysis

Transcriptome sequencing data derived from 79 patients diagnosed with HER2‐enriched breast cancer were retrieved from the TCGA database. Classification of the subtypes for these cases was completed via TCGAbiolinks. The original data are standardized through log (*x*+1). Correlation analysis is implemented by the cor.test function. The matching of EMP3‐related genes and the PI3K–AKT signal pathway is completed on the KEGG official website. The gene set of the PI3K–AKT signal pathway used for gene set enrichment analysis (GSEA) is obtained from the official GSEA website and then executed in GSEA software (4.1.0).

### 2.11. Statistical Analysis

Statistical analyses were performed using SPSS 21.0 and R 4.0.3; statistical significance was defined as a *p* value less than 0.05. A Chi‐square test (*χ* ^2^) was used to analyze the association between clinicopathological variables and two groups of EMP3 expression, namely the negative and positive expression groups. For multivariate analysis, Cox regression analysis was used. Survival analysis was carried out using Kaplan–Meier combined with the log‐rank test. Based on the results of the multivariate Cox regression analysis, T‐stage, N‐stage, EMP3 expression, and linear prediction were used to construct a nomogram with “rms” and “survival” in the R package. Subsequently, the performance of the developed nomogram was rigorously evaluated using both discrimination and calibration analyses. The discrimination capacity of this nomogram was measured by the calculation of either the receiver operating characteristic curve area (AUC) or Harrell’s concordance statistic (*C*‐index), with a *C*‐index between 0.7 and 0.9 considered moderate discrimination. Calibration curves were carried out to compare the nomogram‐predicted DFS probability and observed DFS. We utilized receiver operating characteristics (ROC) curves for 1‐year and 3‐year DFS, which were generated to conduct comparative analyses between the proposed prediction model and the independent prognostic factors of T‐stage, N‐stage, and EMP3 expression.

## 3. Results

### 3.1. Patient Characteristics

This research enrolled 90 patients who received surgical treatment at Cancer Hospital (Henan) throughout the period from January to December 2017 (Figure 1(a)). The average age across all enrolled patients was 50.0 ± 8.6 years, with ages ranging between 26 and 68 years, while 54.4% of the group was premenopause. Most tumors were T1 and T2 (83.3%); 2 cases of Tis were included without lymph node metastasis (LNM) (42.2%). Distant metastases were excluded if present at the time of surgery. The median follow‐up period stood at 54 months, covering a range from 1 to 59 months. By the time of data analysis, 16 enrolled patients had developed recurrence or metastatic events; the DFS was 82.2%.

FIGURE 1Patient flow diagram and immunohistochemical staining for EMP3. (a) Patient flow diagram. (b) Immunohistochemical staining for EMP3 with different intensity. The staining is graded as negative (−), weak (+), moderate (++), and strong (+++).

**Figure:**
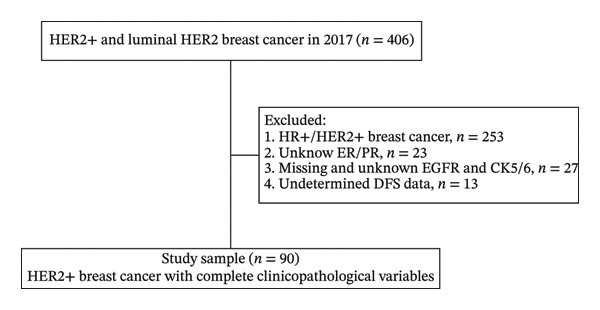
(a)

**Figure:**
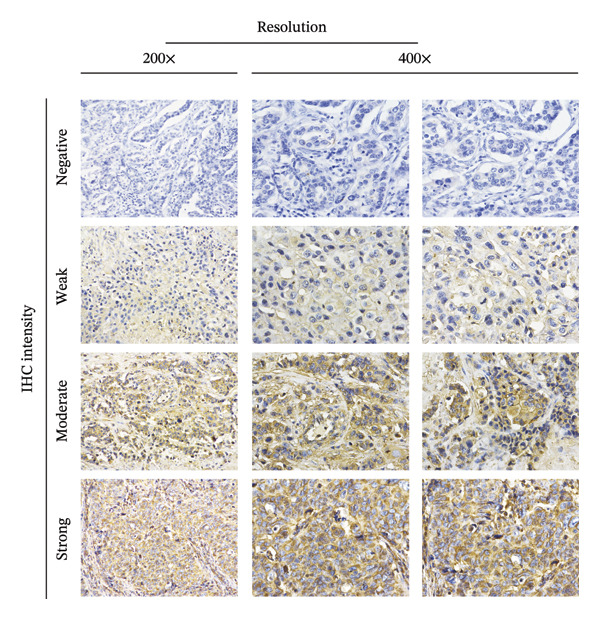
(b)

### 3.2. Association Between EMP3 and Clinicopathological Variables of HER2‐Enriched Breast Cancer

Based on the outcomes derived from IHC (Figure 1(b)), positive expression of EMP3 was detected in 50.0% of patients, while negative expression was detected in the other half (Table 1). The correlation between EMP3 and clinical parameters was evaluated in this research. According to our classification, high EMP3 expression levels showed no notable correlation with patient age or menopausal status or T‐stage, N‐stage, as well as expression of prognostic markers including AR, Ki67, epidermal growth factor receptor (EGFR), and cytokeratin 5/6 (CK5/6).

**TABLE 1:** Tumor characteristics and EMP3 distribution.

Variables	No. (%)/mean ± SD			
	Total (*N* = 90)	EMP3‐negative (*N* = 45)	EMP3‐positive (*N* = 45)	*p*
Age (years)	50.0 ± 8.6	51.1 ± 7.6	48.8 ± 9.5	
< 50	43 (47.8)	17 (39.5)	26 (60.5)	0.058
≥ 50	47 (52.2)	28 (60.0)	19 (40.0)	
Menopause status				
Premenopause	49 (54.4)	22 (44.9)	27 (55.1)	0.290
Postmenopause	41 (45.6)	23 (56.1)	18 (43.9)	
T‐stage				
Tis + T1 + T2	75 (83.3)	38 (50.7)	37 (49.3)	0.777
T3 + T4	15 (16.7)	7 (46.7)	8 (53.3)	
N‐stage				
N0 + N1	64 (71.1)	28 (43.8)	36 (56.2)	0.063
N2 + N3	26 (28.9)	17 (65.4)	9 (34.6)	
Androgen receptor				
Negative	13 (14.4)	9 (69.2)	4 (30.8)	0.134
Positive	77 (85.6)	36 (46.8)	41 (53.2)	
Ki67				
< 30%	13 (14.4)	8 (61.5)	5 (38.5)	0.368
≥ 30%	77 (85.6)	37 (48.1)	40 (51.9)	
EGFR				
Negative	45 (50.0)	22 (48.9)	23 (51.1)	0.833
Positive	45 (50.0)	23 (51.1)	22 (48.9)	
CK5/6				
Negative	78 (86.7)	39 (50.0)	39 (50.0)	1.000
Positive	12 (13.3)	6 (50.0)	6 (50.0)	
NAC				
NO	70 (77.8)	35 (50.0)	35 (50.0)	1.000
YES	20 (22.2)	10 (50.0)	10 (50.0)	

*Note:* Data are expressed as the patient number (%) or mean ± SD. Statistically significant differences were defined as *p* < 0.05. T, tumor; N, lymph node; CK5/6, cytokeratin 5/6; NAC, neoadjuvant chemotherapy.

Abbreviation: EGFR, epidermal growth factor receptor.

### 3.3. High Expression of EMP3 Predicted Worse DFS in HER2‐Enriched Breast Cancer

We analyzed survival‐related endpoints to investigate whether EMP3 could be a viable survival marker. Application of Kaplan–Meier analysis to the enrolled cohort uncovered a tendency toward decreased DFS among patients with elevated EMP3 expression (Log Rank *p* = 0.033) (Figure 2(a)). In stratified analysis, each subgroup showed the same trend, and the N2–N3 subgroup had a significant statistical difference (Figures 2(b), 2(c), 2(d), and 2(e)). In addition, we utilized a model (Cox proportional hazard regression) to identify biomarkers and clinical factors that influence the prognosis status of enrolled patients (Table 2). Univariate statistical analysis verified that EMP3 acted as a notable prognostic factor for DFS among HER2‐enriched breast cancer cases, with a hazard ratio (HR) of 3.209, a 95% confidence interval (CI) ranging from 1.034 to 9.955, and a *p* value of 0.044. At the same time, subsequent multivariate analysis further supplied evidence that EMP3 could serve as an independent prognostic factor, as reflected by an HR of 5.871, a 95% CI of 1.729–19.942, and a *p* value of 0.005. With respect to the other clinicopathological parameters, T‐stage and N‐stage were identified as prognostic indicators for DFS in the univariate model. Multivariate analysis also proved that the T‐stage and N‐stage were significant independent prognostic factors for DFS in HER2‐enriched breast cancer.

FIGURE 2Kaplan–Meier survival curves of DFS between EMP3‐negative and EMP3‐positive groups in HER2‐enriched breast cancer. (a) All patients. (b) Patients with clinical stages N0 and N1. (c) Patients with clinical stages N2 and N3. (d) Patients with clinical stages T1 and T2. (e) Patients with clinical stages T3 and T4. Statistically significant differences were defined as *p* < 0.05. Abbreviations: DFS, disease‐free survival.

**Figure:**
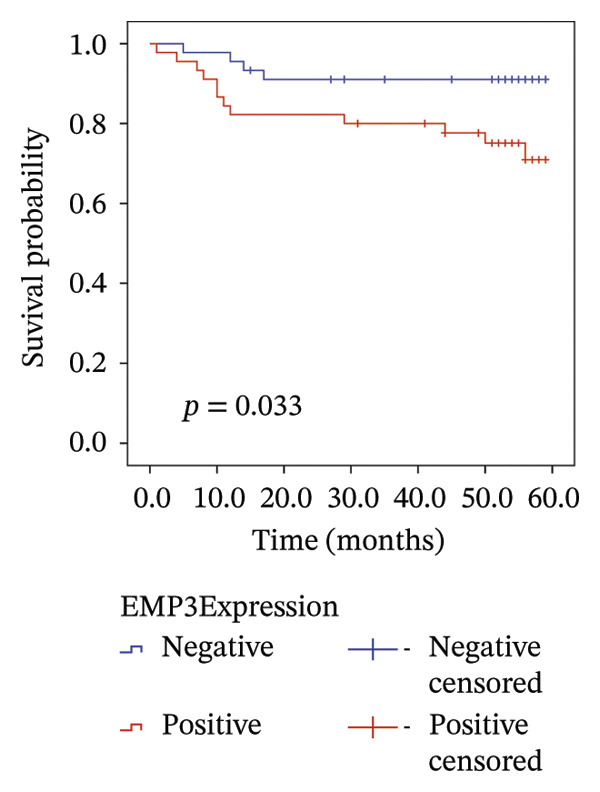
(a)

**Figure:**
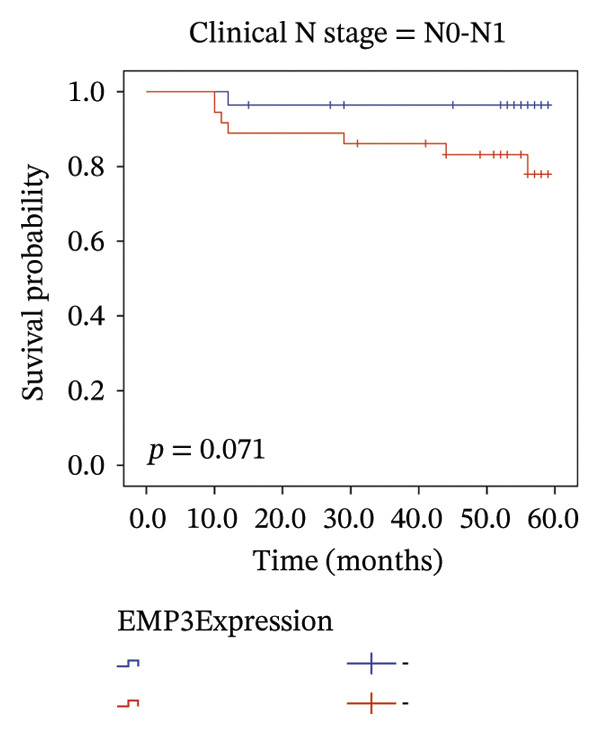
(b)

**Figure:**
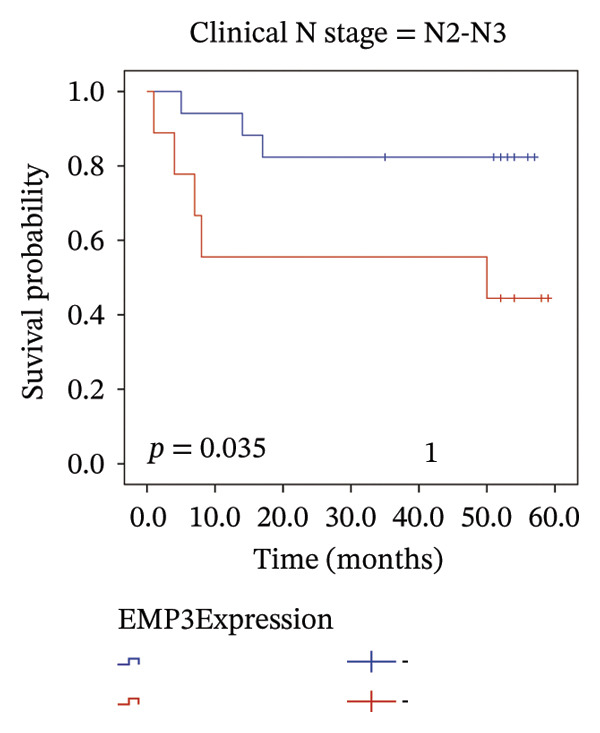
(c)

**Figure:**
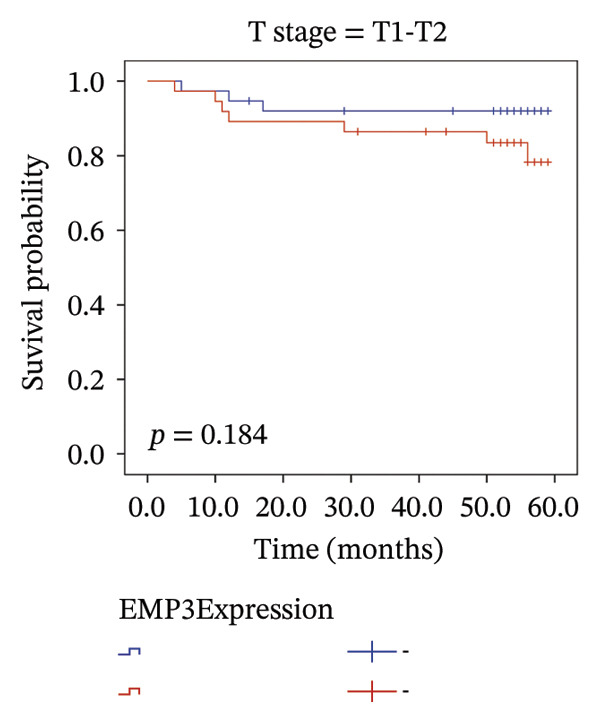
(d)

**Figure:**
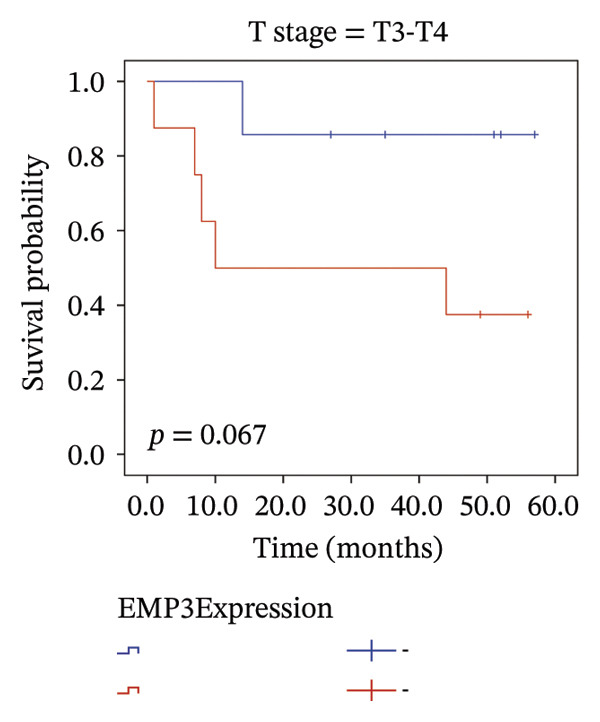
(e)

**TABLE 2:** Univariable and multivariable analyses for predictive factors of DFS.

Variables	Univariate analysis			Multivariate analysis		
	HR	95% CI	*p*	HR	95% CI	*p*
Age (≥ 50 vs. < 50)	1.592	0.578–4.387	0.368			
Menopausal status (postmenopause vs. premenopause)	0.910	0.339–2.444	0.852			
T‐stage (T3 + T4 vs. Tis + T1 + T2)	3.889	1.407–10.751	0.009	4.265	1.488–12.227	0.007
N‐stage (N2–N3 vs. N0–N1)	2.884	1.081–7.691	0.034	4.703	1.639–13.496	0.004
Androgen receptors (positive vs. negative)	0.654	0.186–2.297	0.508			
Ki67 (≥ 30% vs. < 30%)	2.708	0.358–20.505	0.335			
EGFR (positive vs. negative)	1.310	0.488–3.517	0.593			
CK5/6 (positive vs. negative)	1.664	0.474–5.847	0.427			
NAC (yes vs. no)	2.426	0.880–6.684	0.087			
EMP3 (positive vs. negative)	3.209	1.034–9.955	0.044	5.871	1.729–19.942	0.005

*Note:* T, tumor; N, lymph node; NAC, neoadjuvant chemotherapy.

Abbreviations: CI, confidence interval; EGFR, epidermal growth factor receptor; EMP3, epithelial membrane protein 3; HR, hazard ratio.

### 3.4. Prognostic Nomogram for DFS

A predictive nomogram was developed to estimate the probability of DFS using the following three factors: EMP3 expression, T‐stage, and N‐stage (Figure 3(a)). Based on the Cox regression model, we derived the corresponding predictive probability formula: Risk score = exp (1.77 × EMP3 expression + 1.45 × T‐stage + 1.55 × N‐stage). We first drew a vertical line from every single variable to the point scale on the top axis, after which the three acquired point values were added together; a final vertical line was drawn from the total points scale to the 1‐year and 3‐year DFS scales to gain the probability of 1‐year or 3‐year DFS.

FIGURE 3Establishment of nomogram and its calibration and verification. (a) Nomogram for predicting the 1‐ and 3‐year DFS of HER2‐enriched breast cancer. (b) The calibration curves for predicting the 1‐ and 3‐year DFS of HER2‐enriched breast cancer. (c–e) Receiver operating characteristics curve (ROC) comparison of DFS nomogram. Nomogram‐predicted DFS is plotted on the *x*‐axis; actual DFS is plotted on the *y*‐axis.

**Figure:**
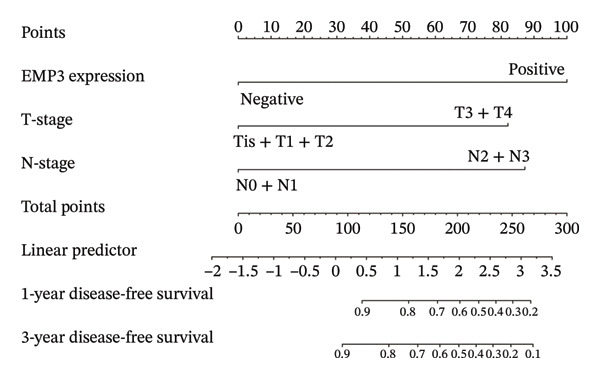
(a)

**Figure:**
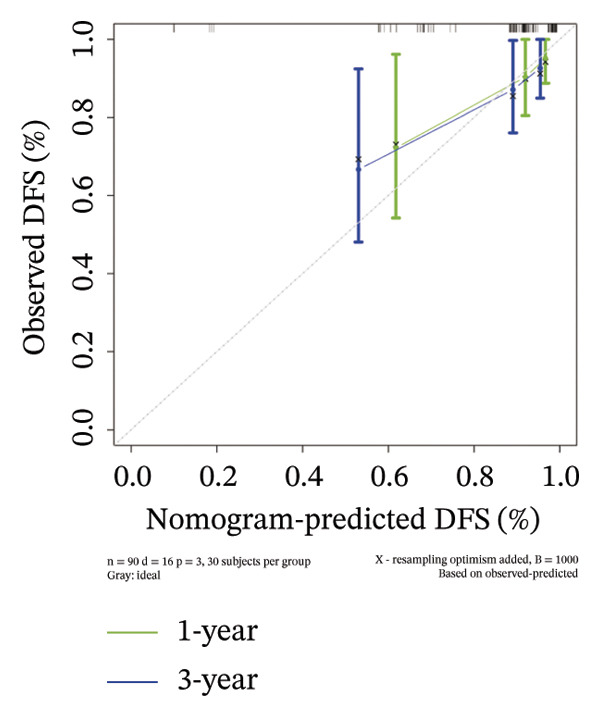
(b)

**Figure:**
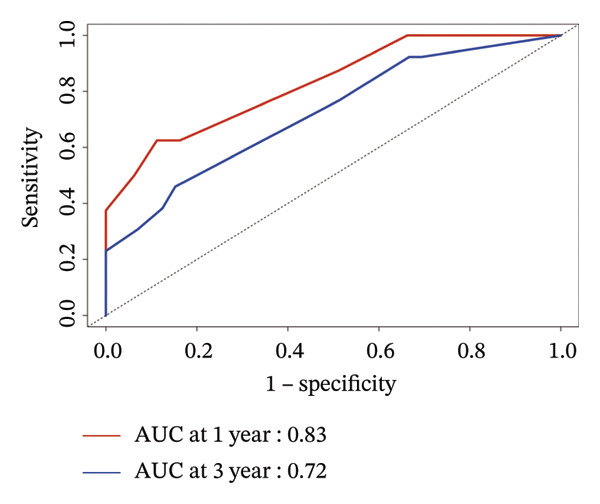
(c)

**Figure:**
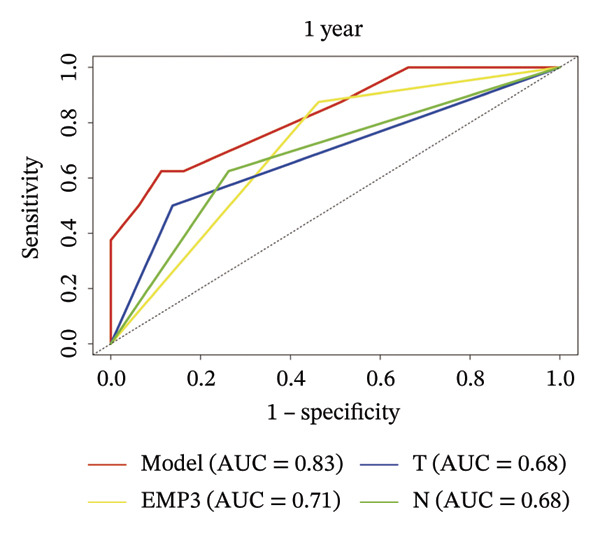
(d)

**Figure:**
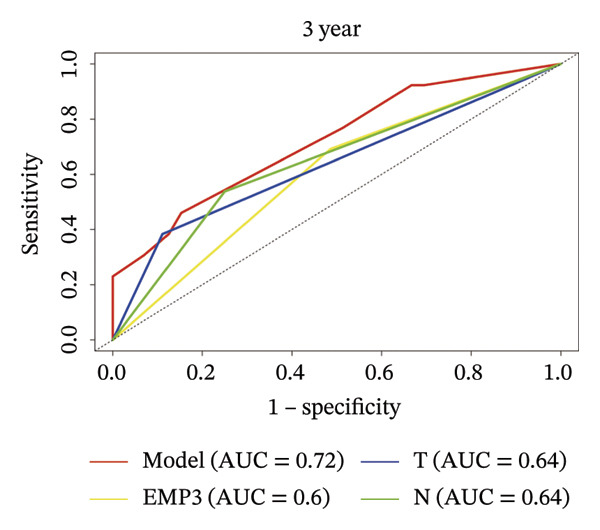
(e)

### 3.5. Calibration and Validation of the Nomogram

This nomogram yielded a *C*‐index value of 0.745, with its 95% CI falling between 0.6076 and 0.8435. Calibration curves demonstrated outstanding agreement between the nomogram prediction and actual observation for 1‐ and 3‐year DFS in both cohorts (Figure 3(b)). Analysis of the ROC curve revealed that the AUC corresponding to the established model was 0.83 in 1 year and 0.72 in 3 years (Figure 3(c)). The comprehensive forecasting ability of 1‐year and 3‐year models is stronger than that of a single index (Figures 3(d), and 3(e)).

### 3.6. EMP3 Expression is Linked to PI3K–AKT Signaling Pathways Within the Transcriptomic Profiles Retrieved From the TCGA

For a more thorough understanding of resistance mechanisms driven by EMP3 in HER2‐enriched breast cancer, we conducted bioinformatics analysis based on transcriptomic data obtained from 79 HER2‐enriched breast cancer patients in the TCGA database. Earlier research has indicated that excessive EMP3 expression in bladder epithelial carcinoma can induce the expression of PI3K *α* 110 and the activity of phosphorylated AKT to be significantly upregulated. The PI3K–AKT pathway activation acts as the most prevalent mechanism responsible for trastuzumab resistance. Therefore, we first screened out genes showing a correlation coefficient exceeding 0.6 relative to EMP3 expression by R software and matched them with PI3K–AKT on the KEGG official website [34]. As shown in Figure 4, 27 genes were consistent with the PI3K–AKT pathway. Furthermore, the correlation coefficient of AKT3 is 0.54, which also shows a moderate correlation.

FIGURE 4The coincidence results of genes that are positively related to EMP3 expression (correlation coefficient greater than 0.6) in the PI3K–AKT signaling pathway. (a) 63 genes with a correlation coefficient greater than 0.6 with EMP3 were used to match the PI3K–AKT pathway [35]. The red box indicates genes that overlap, and the green box indicates genes that do not overlap. (b) 27 gene names that overlap with the PI3K–AKT pathway. (c) Gene set enrichment analysis between the EMP3 high expression group and the low expression group. (d) Heatmap of gene set enrichment analysis.

**Figure:**
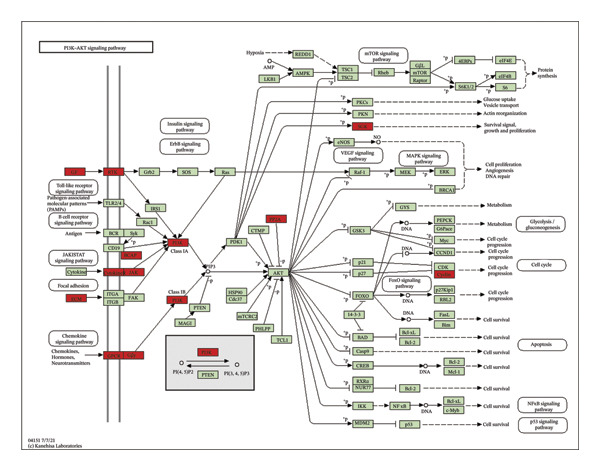
(a)

**Figure:**
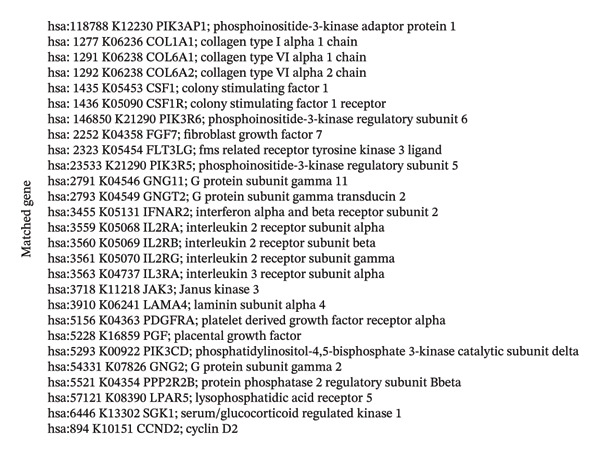
(b)

**Figure:**
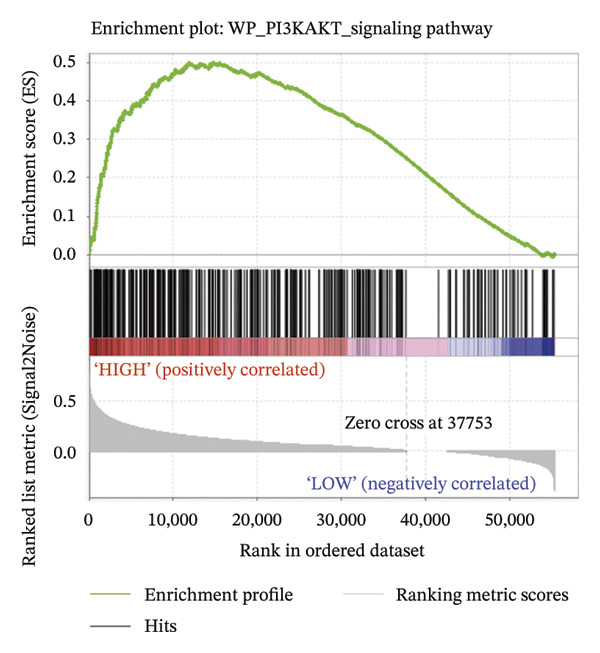
(c)

**Figure:**
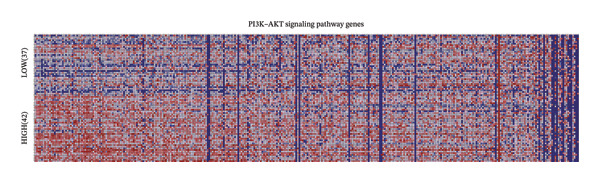
(d)

To further investigate the relationship between EMP3 expression and PI3K–AKT pathway activation, we stratified 79 enrolled patients into two subgroups based on the mean EMP3 expression value: high expression (42 patients) and low expression (37 patients). The two groups were analyzed by GSEA [36]. The GSEA analysis results were shown in Figure 4(c), and the pathway exhibited markedly stronger activation within the EMP3 high‐expression group (Figure 4(d)). The analyses described above suggest that EMP3 is most likely to induce trastuzumab resistance in HER2‐enriched breast cancer through the pathway.

### 3.7. EMP3 Regulates PI3K–AKT Pathway and Enhances Trastuzumab Resistance

To investigate the function of EMP3 in regulating the PI3K–AKT signaling cascade, we performed EMP3 knockdown and overexpression experiments using HER2‐positive cell lines and evaluated the expression levels of downstream signaling markers. As shown in Figure 5(a), following EMP3 knockdown, total protein levels remained unchanged, whereas the expression levels of phosphorylated PI3K, AKT, and mTOR were significantly reduced. In contrast, EMP3 overexpression led to an increase in the phosphorylation levels of these proteins. These findings suggest that EMP3 plays a regulatory role in the activation of this signaling pathway.

FIGURE 5EMP3 regulates the PI3K–AKT pathway and enhances trastuzumab resistance. (a) EMP3, PI3K, AKT, and mTOR gene expressions at the protein level in SK‐BR‐3 cells were validated by western blotting. (b and c) The colony formation assay evaluated the proliferative capacity of cells with differential EMP3 expression under trastuzumab treatment. (d–g) EdU assay evaluated proliferative capacity of cells with differential EMP3 expression under trastuzumab treatment. (h and i) Statistical analysis of the positive rate of EdU. All data were representative of at least three independent experiments. Data are presented as mean ± SD (*n* = 3; unpaired *t*‐test; ^∗∗∗∗^ *p* < 0.0001, ^∗^ *p* < 0.05).

**Figure:**
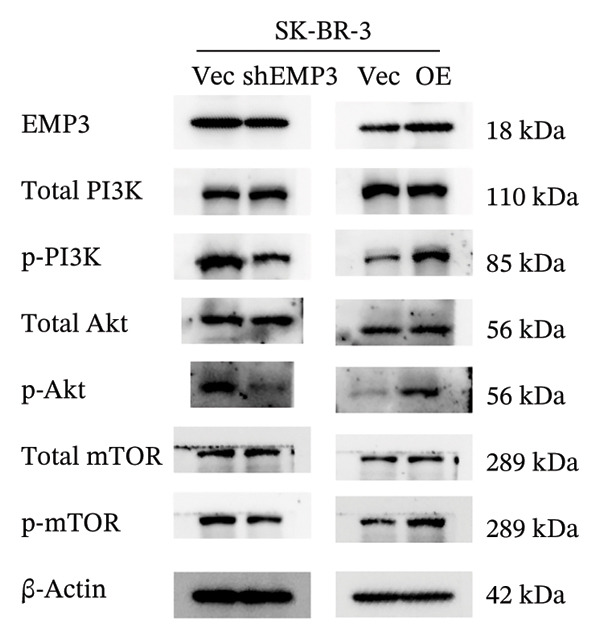
(a)

**Figure:**
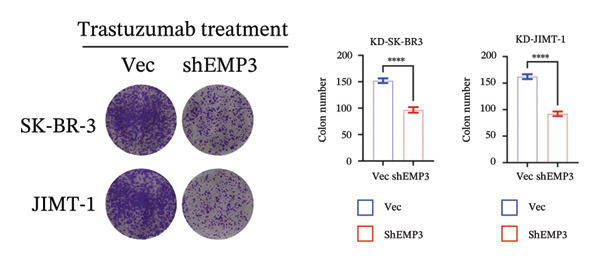
(b)

**Figure:**
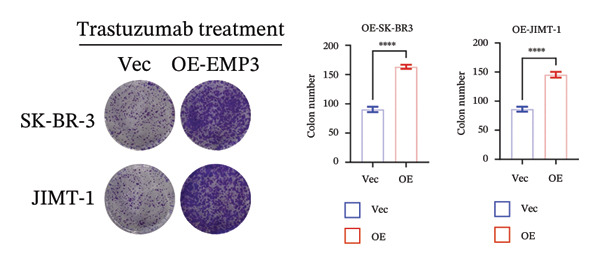
(c)

**Figure:**
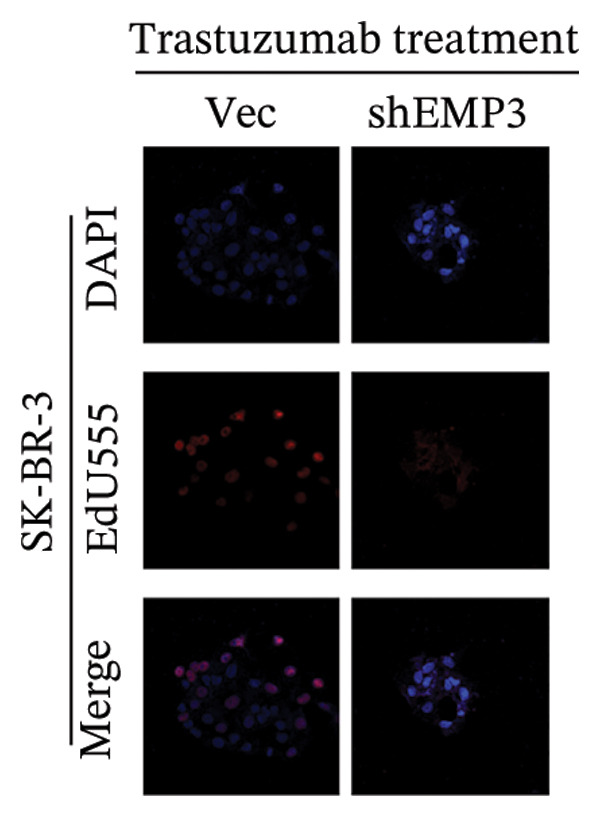
(d)

**Figure:**
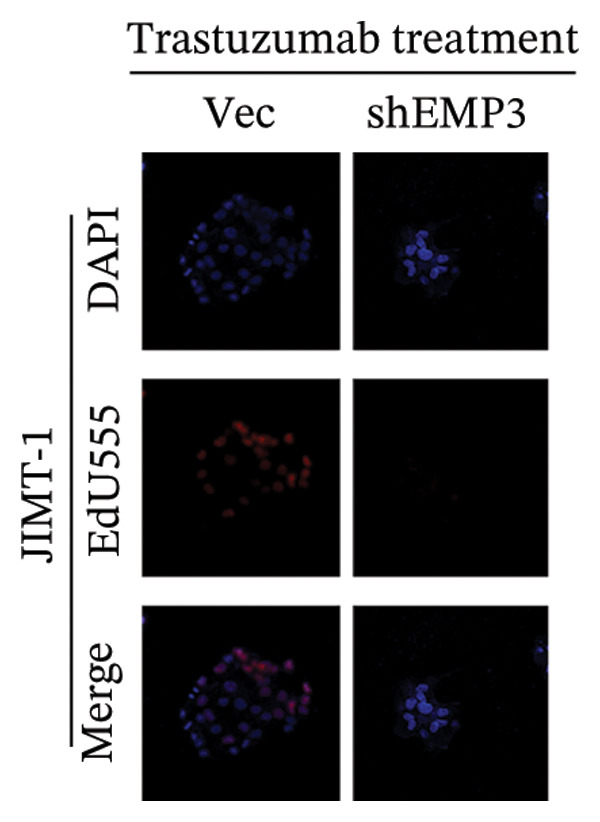
(e)

**Figure:**
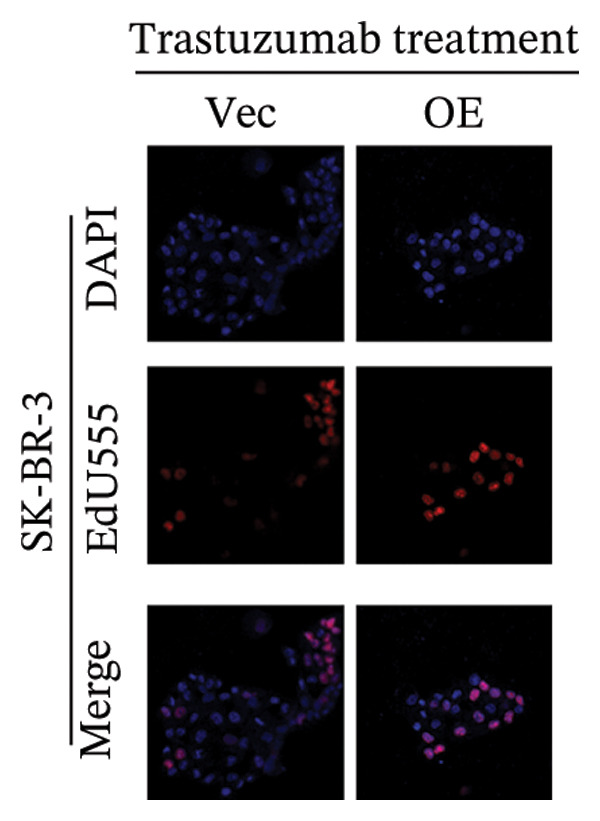
(f)

**Figure:**
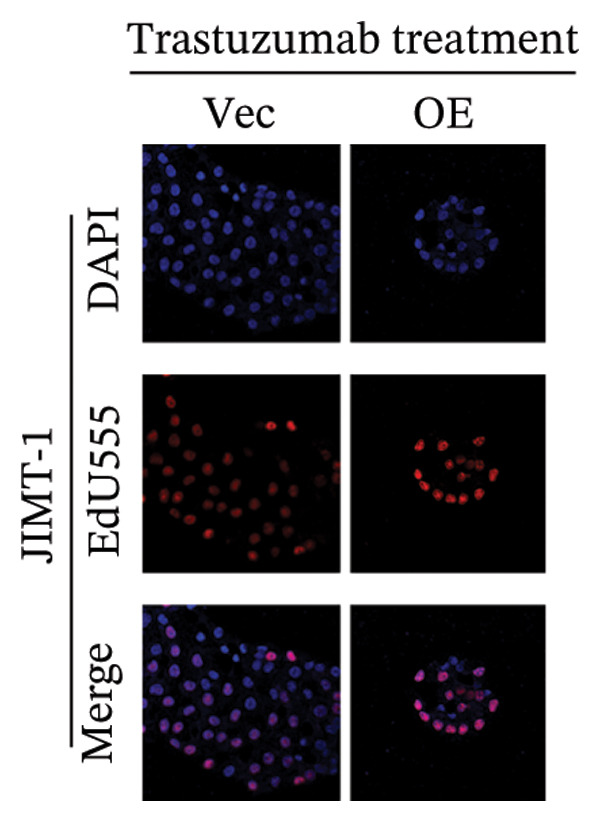
(g)

**Figure:**
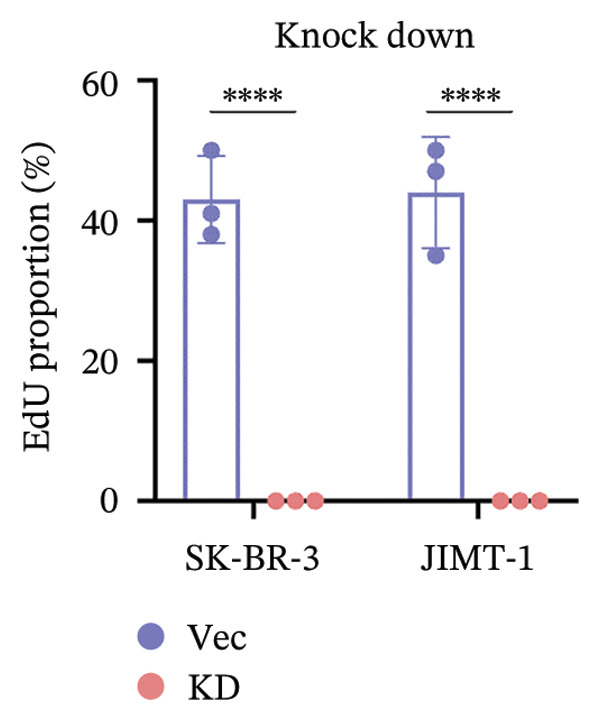
(h)

**Figure:**
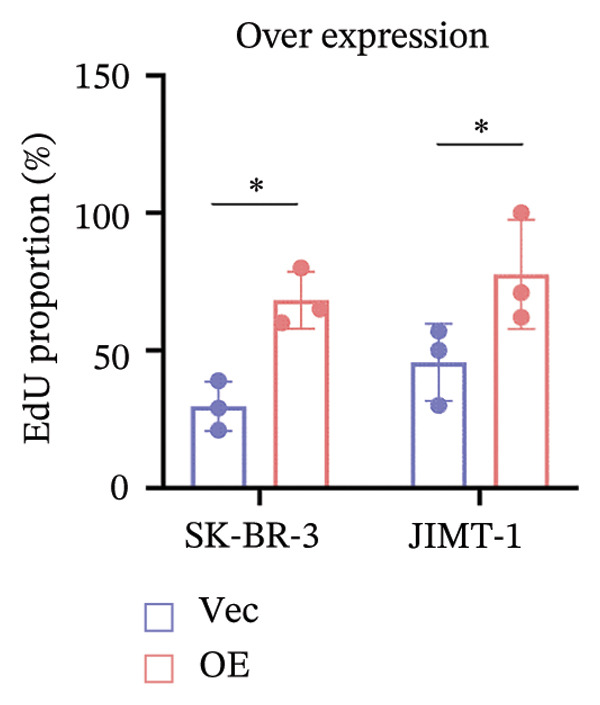
(i)

Given that EMP3 expression correlates with sensitivity to trastuzumab in urothelial carcinoma, we further investigate this relationship by examined the impact of EMP3 expression on trastuzumab sensitivity in the HER2 subtype using proliferation assays and in vivo experiments. Clonogenic and EdU proliferation assays demonstrated that, in the presence of trastuzumab, knockdown of EMP3 enhanced sensitivity to the drug in trastuzumab‐sensitive as well as trastuzumab‐resistant cell lines, whereas overexpression of EMP3 increased resistance (Figures 5(b), 5(c), 5(d), 5(e), 5(f), 5(g), 5(h), and 5(i)). In vivo experiments further support the aforementioned conclusion, consistently detected in sensitive and resistant models (Figure 6).

FIGURE 6In vivo investigation of EMP3 expression and its association with trastuzumab sensitivity. (a) Flowchart of in vivo experiments. (b and c) Changes in tumor volume and weight in the trastuzumab‐sensitive model. (d and e) Changes in tumor volume and weight in the trastuzumab‐resistant model. All data were representative of at least three independent experiments. Data are presented as mean ± SD (one‐way analysis of variance and two‐way analysis of variance; ^∗∗∗∗^ *p* < 0.0001, ^∗∗∗^ *p* < 0.001, ^∗∗^ *p* < 0.01, ^∗^ *p* < 0.05).

**Figure:**
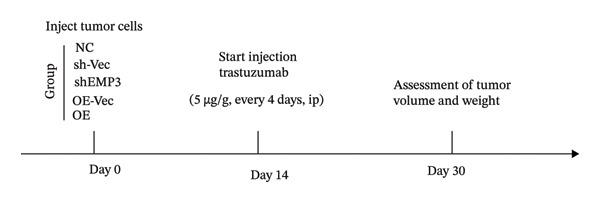
(a)

**Figure:**
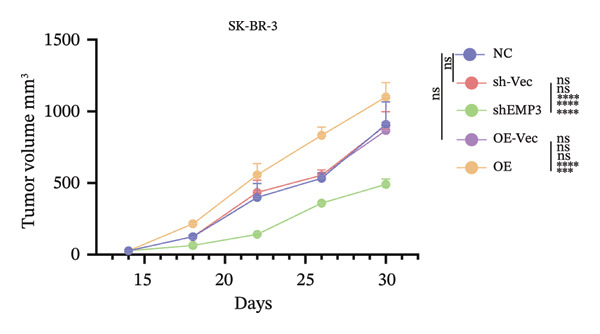
(b)

**Figure:**
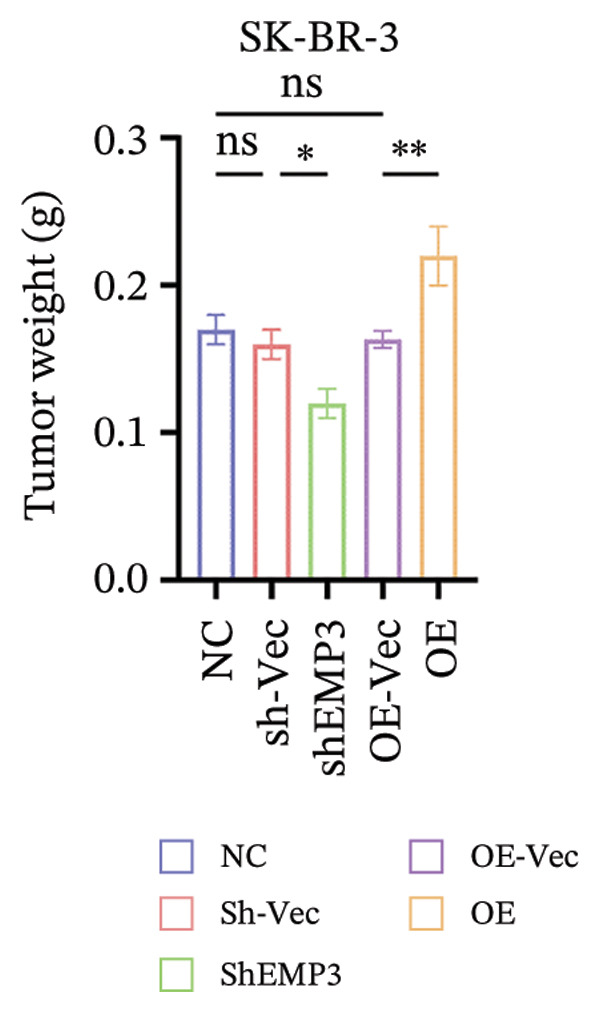
(c)

**Figure:**
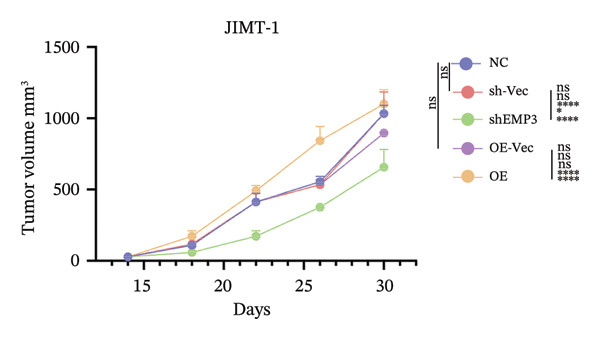
(d)

**Figure:**
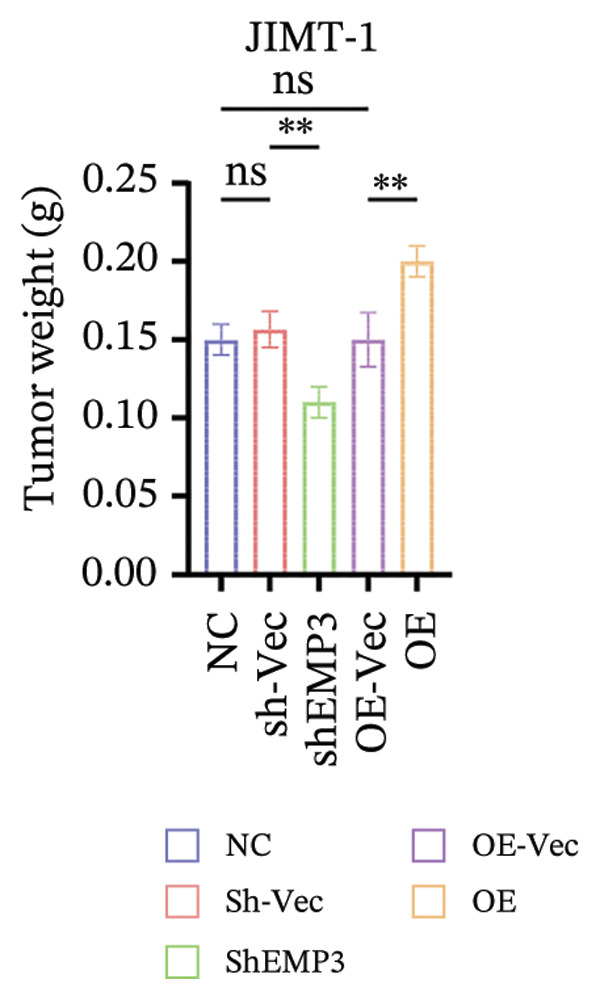
(e)

## 4. Discussion

Though the introduction of trastuzumab has drastically improved the therapeutic outcomes for HER2‐positive breast cancer, primary and secondary trastuzumab resistance still plagues patients suffering from locally advanced or metastatic HER2‐enriched breast cancer due to the lack of powerful combination therapy targets. Thus, in the entire cohort of breast cancer patients, trastuzumab‐resistant recurrent and metastatic HER2‐enriched breast cancers have a very poor prognosis. In this study, we revealed a significant association between EMP3 expression status and the survival profiles of HER2‐enriched breast cancer. In our cohort, high EMP3 expression predicted a decrease in DFS. Transcriptome‐based bioinformatics assessments indicated that EMP3 expression correlated with the PI3K–AKT pathway activation in HER2‐enriched breast cancer patients, which ranks among the major mechanisms underlying trastuzumab resistance. Though Zhou et al. found that EMP3 knockdown triggered phosphorylation of AKT, P70, and mTOR, overexpression inhibited the phosphorylation of these proteins, which implies that EMP3 exerts regulatory effects on the AKT–mTOR signaling pathway. However, the cell lines selected for that research were triple‐negative breast cancer strains, so a similar effect in HER2‐enriched breast cancer cannot be demonstrated.

The molecular basis of functional crosstalk between EMP3 and HER2 remains poorly defined. Structural analyses indicate no significant sequence or domain‐level homology between the two proteins, and their canonical biological roles are distinct. Notably, convergent regulation of the PI3K–AKT signaling pathway by both EMP3 and HER2 suggests a potential compensatory or cooperative mechanism in breast cancer pathogenesis.

To sum up, across our cohort of HER2‐enriched breast cancer patients undergoing follow‐up (long‐term), we measured EMP3 expression and verified that it is associated with the prognosis of the subtype, showing a trend to boost tumor aggressiveness. We therefore conclude that EMP3 carries significant prognostic worth in the HER2‐enriched subtype and is closely tied to the worse survival prospects, specifically in the high‐risk patient group with N2 or N3 tumors. While there was no notable statistical difference among the subgroups of N0–N1, T1–T2, and T3–T4 in the stratified analysis, it was very close to the critical value of 0.05. Considering the small sample size, we believe there will be differences in larger samples. Moreover, bioinformatics analysis of TCGA (same‐type cohort) and the relationship between EMP3 expression and PI3K–AKT pathways were verified, suggesting that EMP3 very likely promotes trastuzumab resistance. Given the pressing need for clinicians to identify effective molecular targets, EMP3 may be a potential target for overcoming trastuzumab resistance.

## Funding

This study was supported by Key Specialty (Disease) Project of Fudan University Affiliated Pudong Hospital, Yjzdzk2025‐05.

## Ethics Statement

The study was approved by the Ethics Committee of Henan Cancer Hospital (No. 2017407). We confirm that all methods are implemented in accordance with relevant guidelines and regulations. Written informed consent was obtained from all study participants.

## Conflicts of Interest

The authors declare no conflicts of interest.

## Data Availability

The data that support the findings of this study are openly available in UCSC Xena at https://xenabrowser.net/datapages/?cohort=TCGA%20Breast%20Cancer%20(BRCA)&removeHub=https%3A%2F%2Fxena.treehouse.gi.ucsc.edu%3A443.
